# Modeling and Docking Studies on Novel Mutants (K71L and T204V) of the ATPase Domain of Human Heat Shock 70 kDa Protein 1

**DOI:** 10.3390/ijms15046797

**Published:** 2014-04-22

**Authors:** Asita Elengoe, Mohammed Abu Naser, Salehhuddin Hamdan

**Affiliations:** Faculty of Bioscience and Medical Engineering, Universiti Teknologi Malaysia, Skudai, Johor 81310, Malaysia; E-Mails: abunaser@fbb.utm.my (M.A.N.); saleh65@utm.my (S.H.)

**Keywords:** adenovirus, Hsp70, PROCHECK, ProQ, ERRAT, Verify 3D, ProSA, docking

## Abstract

The purpose of exploring protein interactions between human adenovirus and heat shock protein 70 is to exploit a potentially synergistic interaction to enhance anti-tumoral efficacy and decrease toxicity in cancer treatment. However, the protein interaction of Hsp70 with E1A32 kDa of human adenovirus serotype 5 remains to be elucidated. In this study, two residues of ATPase domain of human heat shock 70 kDa protein 1 (PDB: 1 HJO) were mutated. 3D mutant models (K71L and T204V) using PyMol software were then constructed. The structures were evaluated by PROCHECK, ProQ, ERRAT, Verify 3D and ProSA modules. All evidence suggests that all protein models are acceptable and of good quality. The E1A32 kDa motif was retrieved from UniProt (P03255), as well as subjected to docking interaction with NBD, K71L and T204V, using the Autodock 4.2 program. The best lowest binding energy value of −9.09 kcal/mol was selected for novel T204V. Moreover, the protein-ligand complex structures were validated by RMSD, RMSF, hydrogen bonds and salt bridge analysis. This revealed that the T204V-E1A32 kDa motif complex was the most stable among all three complex structures. This study provides information about the interaction between Hsp70 and the E1A32 kDa motif, which emphasizes future perspectives to design rational drugs and vaccines in cancer therapy.

## 1. Introduction

The current study explores the interaction between human heat shock protein 70 (Hsp70) and adenovirus. Hyperthermia has been explored as an anti-cancer agent for many decades. While the treatment effects of hyperthermia as a single agent are limited, its ability to potentiate the effects of standard chemo radiotherapies has generated lasting interest. Despite the fact that hyperthermia in combination with adenoviral therapy has shown their effectiveness *in vitro*, currently no clinical trials for this combination treatment is underway. Although these oncolytic adenoviruses are promising as anticancer agents, clinical experiences show that these agents alone failed to generate sustained clinical responses or to cause complete tumor regressions. This is because heterogeneity or indeed lack of expression of receptors (coxsackie adenovirus receptor, CAR) and co-receptors (integrin αvβ3 and αvβ5 classes) in tumors could be implicated in the poor efficiency of infectivity by adenovirus. In addition, many tumors cells fail to support adenovirus replication because of its replication deficiency. Traditionally, elevated temperature as a part of the febrile response of humans was thought to inhibit viral replication. However, several investigators suggest that hyperthermia may in fact enhance viral replication, particularly in tumor cells [1]. The E1A gene products of adenovirus are responsible for activation of the Hsp70 and may affect Hsp70 levels during the cell cycle [2]. Hsp70 enhances viral proteins import and colocalizes in the nucleus with E1A. Bacterial DNAJ and DNAK, which are important for bacteriophage DNA replication, may depend on Hsp70 induction, as documented by Wickner *et al.* in 1992 [3]. Hsp40 and Hsp70 induction promotes production of viral proteins for avian adenovirus CELO [4]. Thus, the purpose of investigating the protein interaction between adenovirus and Hsp70 is to exploit a potentially synergistic interaction to enhance anti-tumoral efficacy and decrease toxicity in cancer therapy; but the molecular interaction between Hsp70 with E1A32 kDa of human adenovirus serotype 5 remains to be elucidated. E1A32 kDa protein consists of 289 amino acids. According to experimental studies, mutation of residues 154, 157, 171 and 174 from cysteine to serine causes a loss of E1A transactivation. In addition, mutation of residue 115 (leucine) to alanine causes complete loss of interaction with host ZMYND11, while mutation of leucine (residue 122) to isoleucine abolishes binding to UBE2I.

Hsp70 and other members of the Hsp70 protein family are described as molecular chaperones which help in the non-covalent folding or unfolding and the assembly or disassembly of other protein structures [5–8]. Human Hsp70 contains 640 amino acids and has two major domains. The 44 kDa, 380 amino acid *N*-terminal domain binds and hydrolyzes ATP, whereas the *C*-terminal domain is required for binding peptides and folding non-native polypeptides. Two functionally relevant subdomains can be differentiated within the substrate binding domain (SBD), an 18 kDa peptide-binding domain and a 10 kDa *C*-terminal domain that contains the Glu-Glu-Val-Asp (EEVD) regulatory motif [9]. It has recently been shown that the EEVD motif of the human Hsp70 molecular chaperone regulates ATP hydrolysis [9], and that it interacts with substrates and the co-chaperone, HDJ-1 [10]. Allosteric interaction with the SBD and the interactions with co-chaperones such as Hsp40 and nucleotide exchange factors all critically depend on the conformation of the nucleotide binding domain (NBD), thus affecting the chaperone function of Hsp70.

Since the structure of Hsp70 is yet to be determined experimentally, development of computational three-dimensional model structures of the protein from the sequence and molecular dynamic simulations on the model structure might help in understanding the dynamics of conformational changes and hence the functional mechanism of the NBDs of Hsp70. Understanding the conformational dynamics of the NBD is the key to understanding how the ATPase motor drives the periodic client binding and releasing of the Hsp70 machine. In this study, K71 and T204 of ATPase domain of human heat shock 70 kDa protein 1 (PDB: 1 HJO) involved in playing a vital role in catalytic activity were mutated. Then, we created 3D mutant structures using PyMol software. Furthermore, the docking program (Autodock Version 4.2) has been used to predict the preferred sites of interaction between the NBD, K71L and T204V mutant models with E1A32 kDa motif.

## 2. Results and Discussion

### 2.1. In-Silico Mutagenesis

Residues 71 and 204 of the NBD protein mutated from lysine (basic polar) to leucine (non-polar) and threonine (polar) to valine (non-polar) respectively. These mutations do not change the overall electronic nature of the side chains. The classification of amino acid chemical properties was based on the research done by Biro *et al.* (2003) [11]. Proteins that contain changes in residues may have some effects on the overall structure or function of the protein. Therefore, all mutants were chosen for the subsequent *in-silico* based modeling.

The conserved LYS71 is a catalytically important residue that affects ATP hydrolysis [12]. The proposed mechanism of ATP hydrolysis suggested that the role of LYS71 in accepting a proton from the hydroxide ion or water molecule involved is in-line with a nucleophilic attack [12–15]. The inorganic phosphate group (Pi) is coordinated by a salt bridge with LYS71, hydrogen bonds to THR13 and THR204 and interacts directly with a calcium ion. A water molecule mediates additional interactions with the protein’s main chain at positions 202, 203 and 204. The Pi-binding site is on the protein face opposite the highly conserved GLY32 loop that has been implicated in the binding of nucleotide release factor (GrpE) to the ATPase domain of Hsp40 (DNAK) [16]. Therefore, there are potential channels for Pi exit to the protein surface. However, release of the inorganic phosphate group has been implicated in the conformational transition of Hsp70 molecular chaperone [17]. Phospho-threonine was postulated as an intermediate of ATP hydrolysis. In addition, ATPase activity of Hsp70 initiates viral DNA replication. This has been demonstrated for bacterial DNAJ which stimulates ATPase activity of Hsp70 to start DNA replication of SV40 [18]. Thus, mutational study of these two residues which are important for ATPase activity was carried out.

### 2.2. Physiochemical Characterization

The computed pI value for NBD, K71L and T204V (pI < 7) indicated their acidic character. The extinction coefficient was calculated as 20,525–20,625 M^−1^·cm^−1^ based on the molar extinction coefficient of TYR, TRP and CYS residues. This measure indicates how much light is absorbed by a protein at a particular wave length. On the basis of instability index, Expasy’s ProtParam [19] classified NBD, K71L and T204V proteins as stable (instability index < 40). Instability index relies upon the occurrence of certain dipeptides along the length of the enzyme. The aliphatic index is defined as the relative volume of a protein that is occupied by an aliphatic side chain. An increase in the aliphatic index increases the thermo stability of globular proteins. The very high aliphatic index of all NBD and mutant proteins infers that these proteins may be stable for a wide range of temperatures. The very low grand average of hydropathicity (GRAVY) index (a negative value GRAVY) of NBD and all the mutant proteins infers that these proteins could result in a better interaction with water (hydrophilic in nature) (Table 1). The secondary structure indicates whether a given amino acid lies in a helix, strand or coil. The results from the SOPMA server [20] revealed that alpha helix dominated among secondary structure elements followed by random coils, extended strand and beta turns (Table 2).

### 2.3. Model Simulation and Evaluation

**Figure 1.**
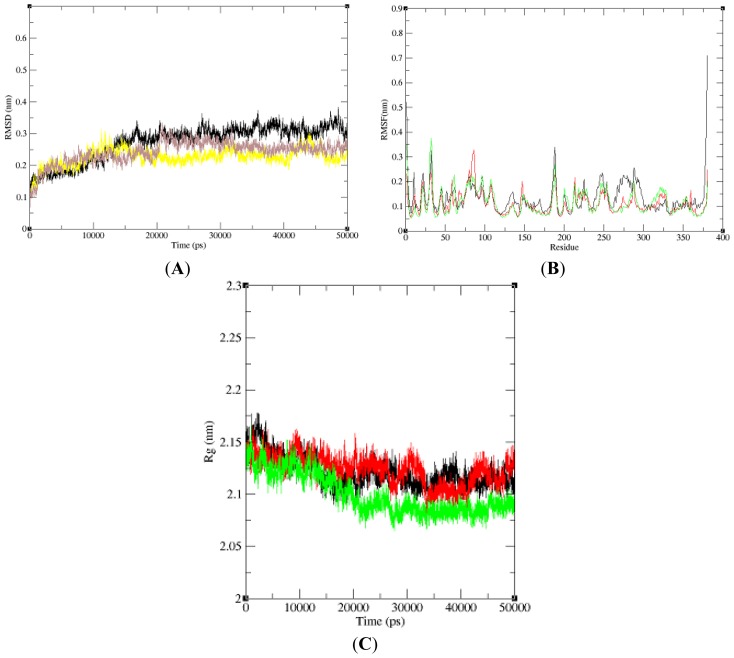
Dynamic changes of the NBD upon mutations. (**A**) Root mean square deviations (RMSD) of NBD (black), K71L (yellow) and T204V (brown); (**B**) Backbone atomic fluctuations (RMSF) of NBD (black), K71L (red) and T204V (green); (**C**) Radius gyration of NBD (black), K71L (red) and T204V (green).

In this paper, we performed three 50 ns (50,000 ps) MD simulations of NBD, K71L and T204V to explore and compare the protein internal dynamics. To analyze the global behavior of the studied systems, the root mean square deviations (RMSDs) of the protein backbone with respect to the initial conformation were plotted *versus* simulation time (Figure 1A). RMSD played an important role in protein stability. The RMSD of the NBD model increased slightly and stabilized at 16,000 ps. K71L RMSD value increased gradually and stabilized at 5000 ps. It decreased slightly and then increased until it stabilized at 12,000 ps. At 40,000 ps, the NBD increased until 45,000 ps and dropped. In T204V’s simulation, the RMSD increased and stabilized at 5000 ps. T204V then increased slightly until it attained a constant level at 21,000 ps; and decreased at 31,000 ps until it stabilized at 35,000 ps. From the RMSD analysis, the T204V structure seemed more stable compared to the K71L and NBD structures due to lower fluctuation of RMSD values from 25,000 to 50,000 ps compared to those of the NBD and K71L. The root mean square fluctuation (RMSF) of the Cα atom of NBD, K71L and T204V as a function of residue number was plotted to evaluate the average fluctuation of each residue during the simulation (Figure 1B). All the residues in mutant models fluctuated around the NBD value which was approximately 0.2 nm. The RMSF of the Cα value of T204V exhibited a higher fluctuation than NBD and K71L at residue 56 (0.88 nm). In addition, NBD and mutant models stabilized at a gyration distance of about 2.11 nm at 20,000 ps (Figure 1C).

**Figure 2.**
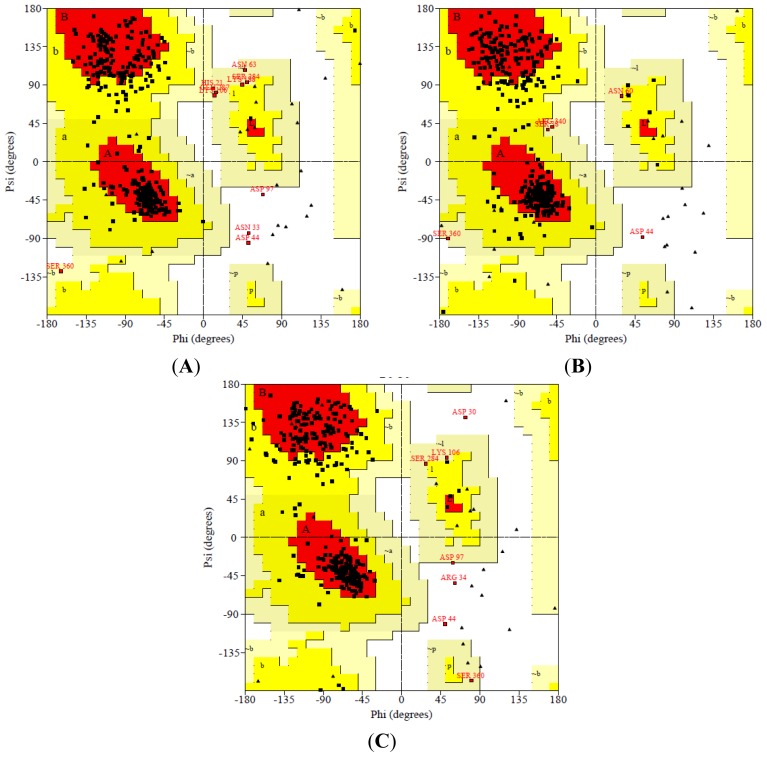
Ramachandran plots generated via PROCHECK for (**A**) NBD protein; (**B**) K71L and (**C**) T204V mutants. PROCHECK shows that the residues in most favored (red), additionally allowed (yellow), generously allowed (pale yellow) and disallowed regions (white color).

After 50,000 ps MD simulation, the geometry of three dimensional protein models was carried out with Ramachandran’s plot calculations using PROCHECK [21]. In the current study, the stereo-chemical evaluation of backbone psi and Phi dihedral angles of the NBD revealed that 81.7%, 15.4%, 2.1% and 0.9% of residues were falling within the most favored regions, additionally allowed regions, generously allowed regions and disallowed regions (ASN33, ASP44 and ASP97) respectively. In general, a score close to 100% implies good stereo-chemical quality of the model [22]. Therefore, these PROCHECK results suggest that the predicted model was of good quality (Figure 2).

The results also show that residues of the NBD model in the most favorable region were more than 80% except K71L and T204V mutants which scored slightly less than 80% for the most favored region (Table 3). However, the stereo chemical quality of the predicted models were found to be satisfactory and a low percentage of residues having phi/psi angles in the outlier region. The analysis explored that no bad contacts and no bad scores for main-chain or side-chain parameters. In spite of that, the overall G-factor values of NBD and mutants were slightly out of range because the values were lower than −0.5 but higher than −1.0. The acceptable values of the G-factor in PROCHECK are between 0 and −0.5, with the best quality models displaying values close to zero [23]. The quality of the protein structures were checked using ProQ [24]. The results show that the predicted LG score (>4: extremely good model) and predicted MaxSub score (>0.5 good model) for all protein models were in an acceptable range of a good model (Table 3).

**Figure 3.**
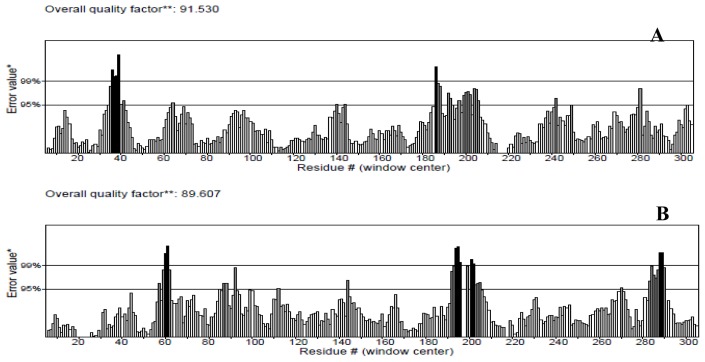

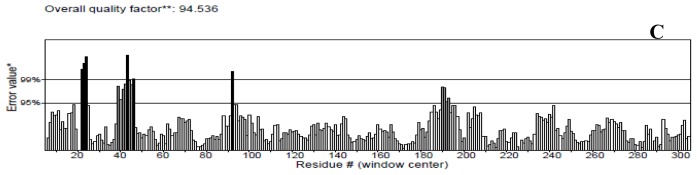
ERRAT plots for (**A**) NBD; (**B**) K71L and (**C**) T204V mutants. Black bars identify the misfolded region located distantly from the active site, gray bars demonstrate the error region between 95% and 99%, and white bars indicate the region with a lower error rate for protein folding. * On the error axis, two lines are drawn to indicate the confidence with which it is possible to reject regions that exceed that error value. ** Expressed as the percentage of the protein for which the calculated error value falls below the 95% rejection limit. Good high resolution structures generally produce values around 95% or higher. For lower resolutions (2.5 to 3 A) the average overall quality factor is around 91%.

The protein structures were also validated by other structure verification servers such as Verify 3D and ERRAT to check the quality of the models. ERRAT works by analyzing the statistics of non-bonded interactions between different atom types, with higher scores indicating higher quality [25]. The ERRAT score for T204V was the highest (94.536%). Nevertheless, this score was close with score values of the NBD (91.530%) and K71L (89.607%) proteins (Figure 3). None of the residues were above the 99% cut off of error-value. However, the generally accepted range is >50 for a high quality model [21]. Thus, this analysis revealed that the backbone conformation and non-bonded interactions of the NBD and mutant models fit well within the range of a high quality model.

**Figure 4.**
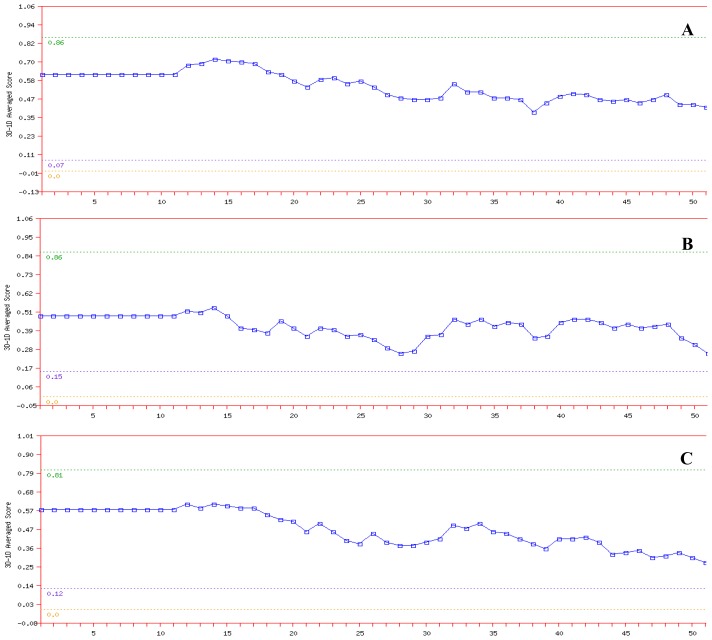
Verify 3D plots for (**A**) NBD protein; (**B**) K71L and (**C**) T204V mutants. Each residue was assigned a structural class based on its location and environment (alpha, beta, loop, polar, nonpolar, *etc*.). A collection of good structures was used as a reference to obtain a score for each of the 20 amino acids in this structural class. The scores of a sliding 21-residue window (from −10 to +10) were added and plotted for individual residues.

In the Verify 3D analysis, it was found that none of the amino acids had a negative score (Figure 4). Therefore, the predicted models were compatible with its amino acid sequence. It should be noted that compatibility scores above zero correspond to an acceptable side chain environment [26].

**Figure 5.**
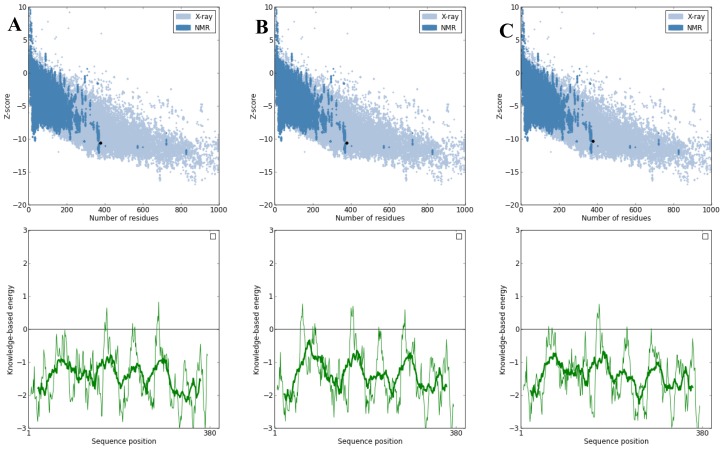
Protein quality scores for (**A**) NBD protein; (**B**) K71L and (**C**) T204V mutants generated through ProSA web server. The results generated display the Z-scores which indicate the overall model quality and energy plots which indicate the local model quality. PROSA-web Z-scores of all protein chains in PDB are determined by X-ray crystallography (light blue) and NMR spectroscopy (dark blue) with respect to their length. The Z-score of protein models were present in the range represented by the large black dot.

PROSA was used to check three dimensional models of proteins under this study for potential errors [27]. The program displays two characteristics of the input structure: its Z-score and a plot of its residue energies. The Z-score indicates overall model quality and measures the deviation of the total energy of the structure with respect to an energy distribution derived from random conformations. Analysis of the K71L and T204V mutants with PROSA showed a Z-Score of −10.38 and −10.63 respectively, indicating no significant deviation from typical native structures of a similar size as the target protein’s Z-Score which was −10.64 (Figure 5). The quality of the protein folds of NBD, K71L and T204V were also evaluated in terms of energy function of amino acid residues. In general, folding energy of the protein showed minimum value as this accounts for the stability and nativity of the molecules. The energy profiles of mutant models in comparison to that of the X-ray structure of the NBD is presented in Figure 5. The energy profile of the K71L and T204V mutant models were consistent with a reliable conformation based on its similarity to that of the NBD.

### 2.4. Active Site Identification

**Figure 6.**
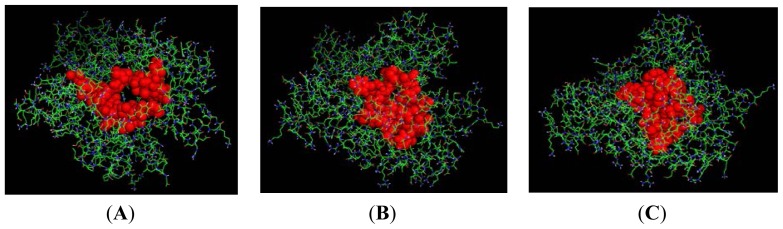
Projection of the predicted active site for (**A**) NBD protein; (**B**) K71L and (**C**) T204V mutants; obtained using Q-SiteFinder web server (shown as red colour).

Size volume, protein volume of active site (Table 4) and the residues forming pocket were obtained using Q-SiteFinder (Figure 6).

### 2.5. Molecular Docking

**Figure 7.**
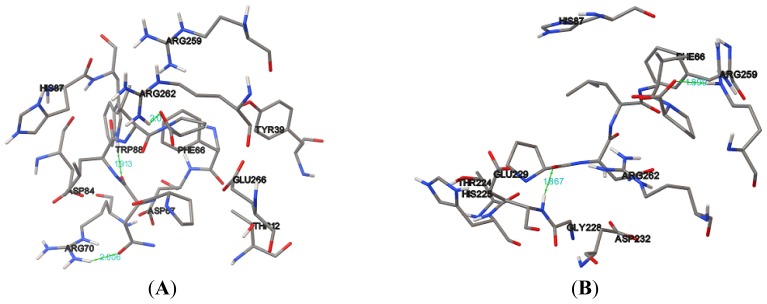

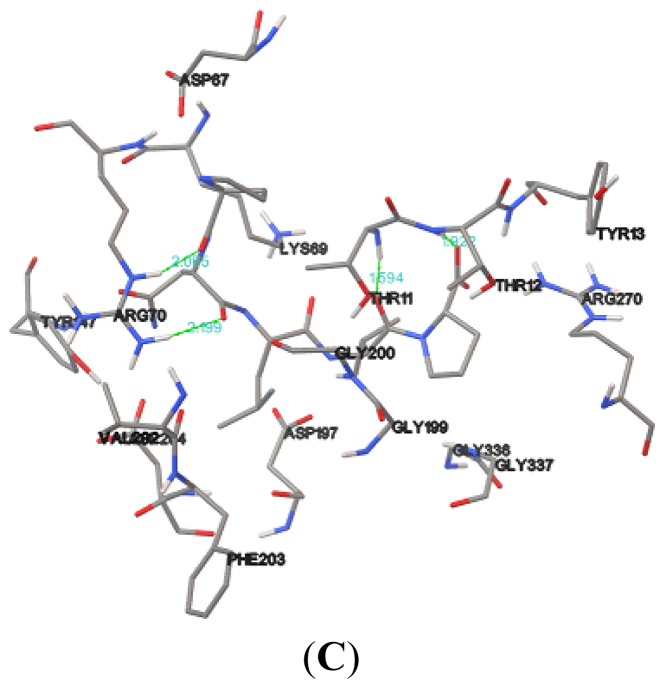
Docking of (**A**) NBD protein; (**B**) K71L and (**C**) T204V mutants with the E1A32 kDa motif. Hydrogen bonds are shown by a green line with its distance (Å).

The negative and low value of Δ*G*_bind_ (−9.09 kcal/mol) indicated strong bonds between T204V and the E1A32 kDa motif, and demonstrated that the protein was in a favorable conformation. Sum of intermolecular energy and torsion energy was the binding energy. Furthermore, the total intermolecular energy of T204V (−12.97 kcal/mol) was found to be lower than the NBD and K71L (−11.93 and −10.64 kcal/mol), stating that the mutant model (T204V) had a better binding affinity than the NBD and K71L in this analysis (Table 5). Hydrogen bonds formed between the compound and the protein usually contribute to the stability of the protein-ligand complexes; a large number of hydrogen bonds form more stable complexes [28,29]. The results show that the NBD, K71L and T204V were stabilized by three, two and four hydrogen bonds with E1A32 kDa motif, respectively (Table 6 and Figure 7). The active residues of the T204V mutant (THR13, THR14 and ARG72) were also involved in the formation of hydrogen bonds, suggesting the protein (T204V) forms a more stable complex than the NBD and K71L (Table 6). Therefore, in the study of protein-ligand binding mechanism, it was revealed that the novel T204V mutant has stronger interaction energy with the E1A32 kDa motif than other protein models.

### 2.6. Model Simulation and Evaluation of Protein-Ligand Complex

**Figure 8.**
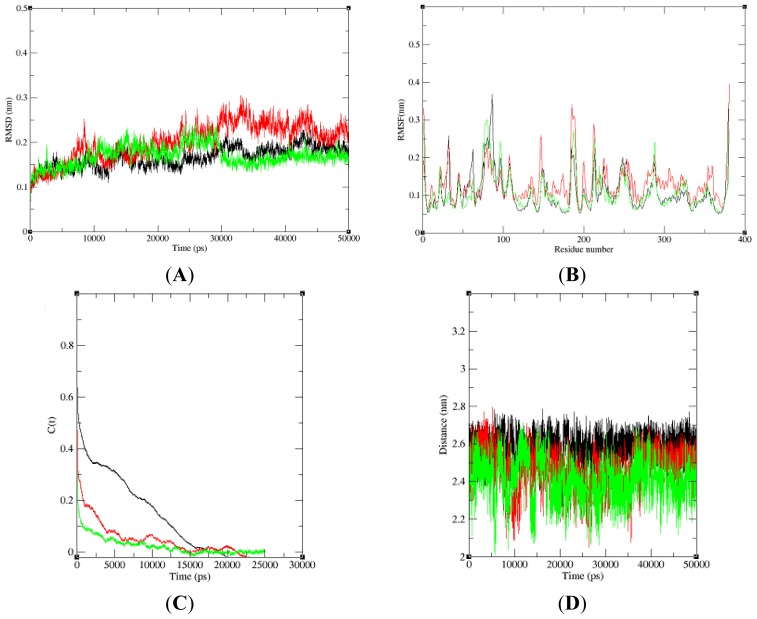
Dynamic changes of the NBD, K71L and T204V-E1A32 kDa motif complex structures. (**A**) Root mean square deviations (RMSD) of NBD (black), K71L (yellow) and T204V (brown); (**B**) Backbone atomic fluctuations (RMSF) of NBD (black), K71L (red) and T204V (green); (**C**) Hydrogen bond autocorrelation of NBD (black), K71L (red) and T204V (green); (**D**) Salt bridge of NBD (black), K71L (red) and T204V (green).

In this study, three 50 ns (50,000 ps) molecular dynamics simulation were run with NBD, K71L and T204V-E1A32 kDa motif complexes. Figure 8A shows the RMSD value of the protein-ligand complex structures over the simulation time. The RMSD value of 0.15 nm for NBD, K71L and T204V-E1A32 kDa motif complex structures were less deviated until 50 ps from their starting structure. NBD-E1A32 kDa motif complex reached stabilization at 35,000 ps (0.17 nm) while the RMSD attained a stable value of 0.20 nm at 35,000 ps for the K71L-E1A32 kDa complex. The T204V-E1A32 kDa complex attained 0.15 nm of RMSD backbone at 35,000 ps during simulation. From these results, it can be concluded that T204V-E1A32 kDa complex deviated less compared to the NBD and K71L-E1A32 kDa complexes indicating that the T204V-E1A32 kDa complex was more stable than the other two complex structures. The RMSF values of carbon alpha for each amino acid residue were obtained from the trajectory data of NBD, K71L and T204V-E1A32 kDa motif complexes shown in Figure 8B. In RMSF analysis, all the residues in the protein model fluctuated between 0.05 and 0.20 nm throughout the simulation period. The NBD-E1A32 kDa motif complex exhibited a high fluctuation up to 0.37 nm at residue 86.

History independent hydrogen bond autocorrelation function was calculated between the protein and the ligand (Figure 8C). The independent autocorrelation function measured the probability of a hydrogen bond present (broken or reformed between a time interval allowed) at time (*t*), given that it was present at time zero. The hydrogen bond analysis revealed that the life-time of a hydrogen bond between protein and ligand is highest for T204V followed by K71L and NBD; the results imply that the association between the protein and the ligand T204V is stronger.

Salt bridges formed between the amino acid side chains at positive ions in NBD, K71L and T204V, and negative ions in the E1A32 kDa motif. The salt bridge is important for stabilizing the protein’s structure. The presence of salt-bridges was a proof of the close proximity in the structure [30]. Salt-bridges occurring between the NBD, K71L and T204V-E1A32 kDa motif were calculated (Figure 8D). All three complexes attained the stable distance of 2.70, 2.60 and 2.40 nm in the entire simulation period. This suggested that the salt bridge with the shortest distance stabilizes the protein the most.

## 3. Experimental Section

### 3.1. Target Sequence

The tertiary structure of the ATPase domain of human heat shock 70 kDa protein 1 was publicly available. The complete amino acid sequence of ATPase domain of human heat shock 70 kDa protein 1, which consists of 380 amino acids, was retrieved from the RCSB Protein Databank (PDB: 1 HJO). The 3D structure of 1 HJO protein viewed using PyMol software [31].

### 3.2. In-Silico Mutagenesis

The functional amino acid residues (K71 and T204) were mutated computationally. These residues played a crucial role in catalytic activity and stabilization of the protein structure. PyMol software was used to alter the amino acid residues.

### 3.3. Physiochemical Characterization

Protein structure analysis was performed using the Expasy’s ProtParam Proteomics server [19]. The secondary structures prediction was carried out using Self Optimized Prediction Method from Alignment (SOPMA) [20].

### 3.4. Model Simulation and Evaluation

The Gromacs package 4.6.3 and the GROMOS 53a6 force field were used to perform MD simulation [32]. In the MD simulations, the NBD and the mutants were simulated using the GROMACS MD simulation software to examine the structural stability at a temperature of 300 K (27 °C). The protein models were solvated in a box of explicit simple point charge (SPC) water molecules and simulated using periodic boundary conditions (PBC) and particle mesh Ewald (PME) summation to take into account the long range electrostatic interactions. One Na+ ion was added to neutralise the total charge of the system for NBD and T204V proteins, meanwhile two sodium ions were added for the K71L protein simulation box. 749, 1004 and 961 steps of steepest descent energy minimization were carried out for NBD, K71L and T204V proteins, respectively. After energy minimization, the system was equilibrated at a constant temperature and pressure for 50 ps. The equilibrated structures were then subjected to molecular dynamic simulations for 50 ns (50,000 ps), LINCS constraint algorithm and 2-fs time step were set to run the simulation. All of the resulting trajectories were analysed using GROMACS utilities. RMSD and RMSF relative to the initial structure were calculated. The stereochemical quality and accuracy of the predicted models were assessed by PROCHECK program [21]. Validation of generated models was further performed by ProQ [24], ERRAT [25], Verify 3D [26] and ProSA [27] programs.

### 3.5. Active Site Identification

The binding sites of the protein were identified using Q-SiteFinder [33,34].

### 3.6. Homology Modeling

The three dimensional model of E1A32 kDa of human adenovirus serotype 5 was not available in the protein database at this time. The complete amino acid sequence of E1A32 kDa was retrieved from UniProtKB (accession number: P03255). BLASTP against the RCSB Protein Databank was carried out to find a suitable template for homology modeling [34]. Crystal structure of (PDB ID: 2 KJE) was selected as a template based on maximum identity with high positives and lower gaps percentage. The percentage of query coverage, sequence identity, positive and gap between the template and target protein were 13%, 100%, 100% and 0% respectively. The three dimensional structure of E1A32 kDa was built using EasyModeller 2.1 software [35], the Graphical User Interface (GUI) of Modeller 9.10, [36] and the model was then viewed using PyMol software. The three dimensional model of the E1A32 kDa motif (PNLVP) was created using the built three-dimensional model of E1A32 kDa as a template. The same homology modeling and 50 ns (50,000 ps) MD simulation approaches were performed before docking with NBD, K71L and T204V.

### 3.7. Molecular Docking

To understand the molecular interactions between E1A32 kDa motif of human adenovirus serotype 5 motif (PNLVP) and the NBD, flexible small molecule rigid protein docking was performed using Autodock Version 4.2 [37]. In the protein, non-polar hydrogen atoms were merged and total Kollman and Gasteiger charge was added to the protein. It was made sure that there were no non-bonded atoms in the protein. Kollman and Gasteiger partial charges were also assigned to the ligand and all torsions were allowed to rotate during docking. NBD and ligand were converted from PDB format to PDBQT format. Residues of the active site were also specified. A grid box was used around the active site to cover the entire protein-binding site and to allow ligands to move freely; and affinity maps of NBD, K71L and T204V (74 × 88 × 108, 70 × 60 × 70, 60 × 70 × 95 containing total grid points of 727,575, 307,501, 411,445 respectively) were calculated using AutoGrid. One hundred Lamarckian Genetic Algorithm (LGA) runs with default parameter settings were performed. Docking was reclustered for 0.5, 1.0 and 2.0 tolerances. The largest docked conformations were clustered at RMS of 1.0 nm and played ranked according to the native Autodock scoring function. The best conformation with the lowest docked energy was chosen from the docking search. The interactions of complex NBD protein-ligand conformations including hydrogen bonds and bond lengths were analyzed. The same docking simulation approach was performed with the single point mutants of NBD (K71L and T204V).

### 3.8. Molecular Dynamics Simulation of Protein-Ligand Complex

The docked complexes of E1A32 kDa motif with NBD and mutants (K71L and T204V) were used as a starting point for MD simulation. The GROMACS package 4.6.3 adopting the GROMOS53a6 force field parameter was carried out to run MD simulation [32]. The protein structures were solvated in cubic box 0.9 nm, using periodic boundary conditions and the SPC (simple point charge) water model. The ligand topology file was generated using the PRODRG server to include heteroatom due to limitations of GROMACS to parameterize the heteroatom group in PDB file [38]. The total charge of the system was neutralized by adding one sodium ion around the molecule for NBD and T204V proteins whereas two sodium ions were added for K71L protein. 993, 945 and 1023 steps of steepest descent energy minimization were carried out for NBD, K71L and T204V proteins respectively. The system was then equilibrated at a constant temperature (303 K) and pressure for 50 ps. Finally, the equilibrated structures were subjected to molecular dynamic simulations for 50 ns (50000 ps) with a LINCS algorithm 2-fs time step. The non-bonded list was generated using an atom-based cut-off of 10 Å. The long range electrostatic interactions were handled by the particle-mesh Ewald algorithm. The trajectory snapshots were taken for structural analysis at every pico-second. RMSD, RMSF, H-bonds and salt bridge formed between the protein and ligand in the docked complex during the simulation were analyzed through Gromacs utilities g_rmsd, g_rmsf, g_hbond and g_salt respectively.

## 4. Conclusions

In this study, we developed 3D mutant models (K71L and T204V), using NBD as a template. The structures were evaluated using RMSD, RMSF analysis, PROCHECK, ProQ, ERRAT, Verify 3D and ProSA programs. All the evidence suggests that the geometric quality of the backbone conformation, energy profile, residue interaction and contact of the predicted 3D structures of K71L and T204V mutants were well within the limits of reliable structures. The protein-protein docking study was elucidated for the purpose of finding protein interaction between Hsp70 and E1A32 kDa of human adenovirus serotype 5 motif; which might help in exploiting a potentially synergistic interaction to enhance anti-tumoral efficacy and decrease toxicity in cancer treatment. The interaction energy of docking between Hsp70 and E1A32 kDa motif was calculated and analyzed using the Autodock 4.2 programme. The results show that the novel T204V mutant was found to have the lowest binding energy (−9.09 kcal/mol) and intermolecular energy (−12.97 kcal/mol) among other protein models. Moreover, the protein-ligand complex structures were validated by RMSD, RMSF, hydrogen bonds and salt bridge analysis. It proved that T204V had strong bonds with E1A32 kDa motif and the complex structure was stable. Therefore, further biochemical and *in vivo* investigations of *in-silico* interpretations of this protein structure will be studied for the development of new therapy for the efficient cancer treatment.

## Figures and Tables

**Table 1.** The physiochemical character of the NBD protein, K71L and T204V mutants as predicted by Expasy’s Prot-Param program.

Protein	Length	M.wt (Daltons)	pI	−R	+R	Extinction coefficient (M^−1^·cm^−1^)	Instability index	Aliphatic index	GRAVY
NBD	380	41,827.4	6.69	50	49	20,525	35.09	88.32	−0.274
K71L	380	41,812.4	6.38	50	48	20,525	34.74	89.34	−0.253
T204V	380	41,825.5	6.69	50	49	20,525	34.99	89.08	−0.261

**Table 2.** Secondary structures of the NBD protein, K71L and T204V mutants.

Secondary structure	Alpha helix(Hh)	Extended strand (Ee)	Beta turn (Tt)	Random coil (Cc)
NBD	42.89	19.74	7.63	29.74
K71L	44.21	18.68	8.16	28.95
T204V	44.47	18.95	6.84	29.74

**Table 3.** Validation of NBD, K71L and T204V mutants using PROCHECK and ProQ.

Structure	Ramachandran plot statistics (%)				Goodness factor			ProQ	
		
	Most favoured	Additionally allowed	Generously allowed	Disallowed	Dihedral angles	Covalent forces	Overall average	LG Score	Max–sub
NBD	81.7	15.4	2.1	0.9	−0.61 *	−0.95 *	−0.66 *	5.707	0.451
K71L	79.9	18.6	0.9	0.6	−0.59 *	−1.02 **	−0.67 *	5.497	0.425
T204V	78.7	19.2	1.2	0.9	−0.61 *	−0.91 *	−0.64 *	5.862	0.424

* Lower than −0.5-unusual;

** Lower than −1.0-highly unusual.

**Table 4.** Predicted active sites of the NBD protein, K71L and T204V mutants.

Protein	Site volume (cubic Å)	Protein volume (cubic Å)	Residues that forming pocket
NBD	434	34357	ASP10,LEU11,GLY12,THR13,THR14,TYR15,PHE68,ASP69,LYS71,ARG72,TRP90,THR145,VAL146,PRO147,ALA148,GLU175,PRO176,ILE197,PHE198,ASP199,GLY201,GLY202,GLY203,THR204,ASP206,VAL207,SER208,THR222,VAL337,VAL369
K71L	496	34418	ASP10,LEU11,GLY12,THR13,PRO14,TYR15,CYS17,ARG72,VAL146,PRO147,ALA148,TYR149,GLU175,PRO176,ALA179,ILE197,PHE198,ASP199,LEU200,GLY201,GLY202,GLY203,THR204,ASP206,VAL207,SER208,THR222,ALA223,GLY224,LYS271,ARG272,VAL337,GLY338,GLY339,GLY340,ALA368,VAL369,ALA370
T204V	532	34224	ASP10,LEU11,GLY12,THR13,THR14,TYR15,SER16,CYS17,LYS71,ARG72,VAL82,THR145,PRO147,ALA148,TYR149,PHE150,GLU175,ASP199,LEU200,GLY201,GLY202,GLY203,VAL204,PHE205,ASP206,ARG272,VAL337,GLY338,GLY339,PRO365,ASP366,GLU367,ALA368,VAL369,ALA370

**Table 5.** Docking results of NBD protein, K71L and T204V mutants with the E1A32 kDa motif.

Protein	NBD	K71L	T204V
Binding energy (kcal/mol)	−8.05	−6.76	−9.09
kI (μM)	1.26	11.04	0.22
Intermolecular Energy (kcal/mol)	−11.93	−10.64	−12.97
Internal energy (kcal/mol)	−2.45	−2.55	−1.49
Torsion energy (kcal/mol)	3.88	3.88	3.88
Unbounded Extended energy (kcal/mol)	−2.45	−2.55	−1.49
Cluster RMS	0.00	0.00	0.00
Reference RMS	86.09	72.93	76.96

**Table 6.** Hydrogen bonds interaction studies of the NBD protein, K71L and T204V mutants with the E1A32 kDa motif.

Protein	Donor atom	Acceptor atom	Distance (Å)
NBD	ARG72:HH21	ASN2:OD1	1.913
	TRP90:HE1	ASN2:O	2.006
	ARG264:HH11	PRO5:O,OXT	2.000

K71L	GLU231:HN	PRO1:O	1.867
	ARG261:HE	PRO5:O	1.899

T204V	THR14:HN	PRO5:O	2.065
	ARG72:HE	PRO:O	2.199
	ARG72:HH21	ASN2:O	1.594
	THR13:HN	VAL4:O	1.922
